# The mechanisms underlying sexual differentiation of behavior and physiology in mammals and birds: relative contributions of sex steroids and sex chromosomes

**DOI:** 10.3389/fnins.2014.00242

**Published:** 2014-08-14

**Authors:** Fumihiko Maekawa, Shinji Tsukahara, Takaharu Kawashima, Keiko Nohara, Hiroko Ohki-Hamazaki

**Affiliations:** ^1^Molecular Toxicology Section, Center for Environmental Health Sciences, National Institute for Environmental StudiesTsukuba, Japan; ^2^Division of Life Science, Graduate School of Science and Engineering, Saitama UniversitySaitama, Japan; ^3^Ecological Genetics Research Section, Center for Environmental Biology and Ecosystem Studies, National Institute for Environmental StudiesTsukuba, Japan; ^4^College of Liberal Arts and Sciences, Kitasato UniversitySagamihara, Japan

**Keywords:** sexual differentiation of the brain, neurosteroids, birds, rodents, chimera

## Abstract

From a classical viewpoint, sex-specific behavior and physiological functions as well as the brain structures of mammals such as rats and mice, have been thought to be influenced by perinatal sex steroids secreted by the gonads. Sex steroids have also been thought to affect the differentiation of the sex-typical behavior of a few members of the avian order Galliformes, including the Japanese quail and chickens, during their development *in ovo*. However, recent mammalian studies that focused on the artificial shuffling or knockout of the sex-determining gene, *Sry*, have revealed that sex chromosomal effects may be associated with particular types of sex-linked differences such as aggression levels, social interaction, and autoimmune diseases, independently of sex steroid-mediated effects. In addition, studies on naturally occurring, rare phenomena such as gynandromorphic birds and experimentally constructed chimeras in which the composition of sex chromosomes in the brain differs from that in the other parts of the body, indicated that sex chromosomes play certain direct roles in the sex-specific differentiation of the gonads and the brain. In this article, we review the relative contributions of sex steroids and sex chromosomes in the determination of brain functions related to sexual behavior and reproductive physiology in mammals and birds.

## Introduction

In most eukaryotic species, sex differences exist within aspects of behavior as well as physiology. Since typical sexual behaviors and reproductive physiology are crucial to produce offspring, it is important to understand the mechanism of sexual differentiation in the brain in conjunction with sex-specific behaviors and physiology. From the standpoint of environmental science, several external factors from the industrialized world can impair reproduction. Studies have shown that the specific chemicals called endocrine disruptors can influence sexual differentiation in many species (Colborn et al., 1993). It has been reported that bisphenol-A and organotin compounds are examples of such endocrine disruptors that mimic and/or antagonize the functions of sex steroids and impair sexual differentiation in wildlife (Flint et al., 2012; Lewis and Ford, 2012). Therefore, research has been conducted on how such chemicals affect sex steroid-dependent organization of various organs, including the brain, during development, in order to protect wildlife and humans from potential endocrine disruptors (Frye et al., 2012). On the other hand, the knowledge regarding sexual differentiation has increased with progress in basic research (Arnold, 2004; Arnold and Chen, 2009) and the endpoints for investigating how environmental chemicals impair normal sexual differentiation might be updated subsequently. In this article, we review the relative contribution of gonadal steroids and sex chromosomes to the differentiation of brain functions related to the sexual behavior and reproductive physiology in mammals and birds by focusing on the recent findings.

## Chromosomal compositions and gonadal sex determination in mammals and birds

The molecular mechanism of gonadal development in mammals and birds has been studied extensively. In most mammals, the males have one X chromosome and one Y chromosome whereas females have two X chromosomes. The gene *Sry/SRY*, located on the Y chromosome has been shown to be critical for testis development in most mammals (Gubbay et al., 1990; Sinclair et al., 1990; Koopman et al., 1991). The mechanism of testis determination in mammals is similar to that of the gonadal sex determination system of some teleost fish whose sex is determined by the specific gene *DMY* located on the Y chromosome (Matsuda et al., 2002). The sex chromosomes of birds are designated Z and W, where male birds have two Z chromosomes, while female birds have one Z chromosome and one W chromosome. The *DMRT1* gene on the Z chromosome, which encodes the doublesex and mab-3-related transcription factor 1 (DMRT1), is required for testis determination in birds of the order *Galliformes* (Smith et al., 2009). Birds lack a global mechanism of dosage compensation to equalize the expression of genes between sexes on the Z chromosome(s). Moreover, male-biased gene expression in gonads has been reported for most genes located on the Z chromosome, with variable levels of expression depending on the locus (Mank and Ellegren, 2009). *DMRT1* in the order *Galliformes* is an example of such a gene and the amount of DMRT1 produced from expression of the single *DMRT1* gene on the single Z chromosome in females is insufficient to induce the formation of a testis. It has also been reported that knockdown of DMRT1 by RNA interference in the male chicken gonadal primordium results in feminization of the gonads (Smith et al., 2009). Therefore, the lower level of *DMRT1* expression presumably induces the gonadal primordium to develop into ovary. Dosage compensation seems to occur on the Z chromosome at a gene-by-gene level in avian organs besides the gonads, such as the brain (Mank and Ellegren, 2009). This fact is the basis for the argument that sex chromosomes autonomously regulate sexual physiology and function in somatic tissues.

## Gonadal hormone-dependent sexual differentiation in the mammalian behavior

In contrast to gonadal development, evidence suggests that sexual behavior is masculinized and defeminized during the perinatal or postnatal period by androgen secreted by the testes, irrespective of *Sry* expression in the brain. The action of testicular androgens in the developing brain is critical to the expression of the male-typical pattern of behaviors in mammals (Phoenix et al., 1959; Arnold and Gorski, 1984). In rodents, testosterone secreted by the testes is locally converted to 17β-estradiol (E2) by the cytochrome P450 enzyme, aromatase, in the brain, and E2 in turn, masculinizes/defeminizes the brain (MacLusky and Naftolin, 1981).

In rat, which is one of the most popular mammalian models for studying sexual differentiation in the brain, testosterone is first detected in the testes on embryonic day 15.5 and testosterone synthesis in the testes peaks at approximately, embryonic day 18.5 (Warren et al., 1973). Testicular testosterone synthesis as well as plasma testosterone concentrations decrease sharply after birth. Since the plasma testosterone level in rats from day 18 of gestation to day 5 postpartum is higher in male than in female rats (Weisz and Ward, 1980), the male brain is exposed to higher levels of testosterone than female brain. Therefore, this perinatal period is considered to be a critical period for sexual differentiation in the rat brain (MacLusky and Naftolin, 1981) and this hypothesis was then supported by the experiments described below.

Injection of female rats with either testosterone propionate (TP) or E2 during the perinatal period resulted in decreased ability to display female sexual behaviors in adulthood (Phoenix et al., 1959). In female mice, knockout of α-fetoprotein that binds to estrogen, thus protecting the developing female brain from exposure to estrogen, has been reported to cause reduction in lordosis, a manifestation of female sexual behavior, and an increase in expression of the male-typical sexual behavior, that is, mounting (Bakker et al., 2006). In addition, it has been reported that estrogen receptor (ER)- α–knockout males exhibited decreased frequency of intromission, another male-typical sexual behavior (Ogawa et al., 1997). Based on these results, the differentiation of stereotypic pattern of sexual behavior in rodents is thought to be, at least in part, due to the effects of sex steroids. Sex-specific copulatory behavior is assumed to be accompanied by structural changes in the brain. Various studies demonstrating morphological sex differences in the brains of rats and mice discussed below.

## Gonadal hormone-dependent sexual dimorphic nuclei in the mammalian brain

Certain brain nuclei exhibit sex differences, in terms of volume and number of neurons and/or synapses and are generally referred to as sexually dimorphic nuclei. Difference in volume of the nuclei is attributable to the differences in number of neurons. Gorski et al. discovered a nucleus in the preoptic region that shows morphological sex differences in rats (Gorski et al., 1978, 1980); this nucleus is now known as the sexually dimorphic nucleus of the preoptic area (SDN-POA), though its physiological functions are still unknown. The SDN-POA is significantly larger and contains more neurons in male than in female rats. Similarly, the volume of the calbindin-D28K-immunoreactive area in the SDN-POA is 2–4 times larger in male than in female rats (Simerly et al., 1997; Sickel and McCarthy, 2000; Orikasa et al., 2007). Moreover, following neonatal castration, the SDN-POA volume in adult male rat decreased (Gorski et al., 1978), whereas injection of TP in females during the early developmental period resulted in an increase in the SDN-POA volume in adult female rats to match that of adult male rats (Gorski et al., 1978; Dohler et al., 1984). On the other hand, injection of TP in adult rats has no effect on the volume of the SDN-POA. These findings suggest that testosterone synthesized in the rat testes affects the brain during development and not during adulthood, to increase the volume of the SDN-POA. Injection of E2 instead of TP in postnatal female rats also increased the size of their SDN-POA during adulthood. Injection of an ER-α agonist, and not an ER-β agonist, mimicked the effect of E2 (Patchev et al., 2004). The effects of estrogen, which is converted from androgen by aromatization and then binds to ER-α during the perinatal period, may be essential to establish the sexual dimorphism in the SDN-POA in rats.

During development, gonadal steroids also influence the sexually dimorphic formation of the principal nucleus of the bed nuclei of the stria terminalis (BNSTp). The BNSTp of the adult male rats is larger and contains more neurons than that of adult females (del Abril et al., 1987; Hines et al., 1992). Perinatal orchidectomy of males and perinatal androgenization of females by injection of TP prevent the occurrence of sexual dimorphism in BNSTp (Guillamon et al., 1988; Chung et al., 2000). Inactivation of androgen receptor results in feminization of the testes, which then show an ovary-like phenotype, and also reduction in the volume of the BNSTp in male rats (Durazzo et al., 2007). Sex differences in the volume and number of neurons in BNSTp do not occur in mice deficient in either aromatase or the ER-α gene, because of feminization of BNSTp in males (Tsukahara et al., 2011). On the other hand, mice deficient in the ER-β gene show sex differences in BNSTp (Tsukahara et al., 2011). These results suggest that estrogen is synthesized from androgen and that its effects, exerted through binding to ER-α during the perinatal and/or adult stage, appear to be involved in the male-typical formation of the BNSTp in mice. It has recently been reported that, in the BNST, gene expression of *Brs3, Cckar* and *Sytl4*, was sexually dimorphic, and regulated female and male sexual behaviors (Xu et al., 2012). However, since expression of these genes was altered by gonadectomy, sexual dimorphism was suspected to be attributable to the effect of sex steroids at the adult stage. More recently, it has been reported that the number of CRH neurons in the BNST (Fukushima et al., 2013) can be altered by TP injection in the perinatal period.

In contrast to the effects of sex steroids on the SDN-POA and BNSTp, the size of the anteroventral periventricular nucleus of POA (AVPV) in rats was reduced by androgens or estrogens during the perinatal period (Ito et al., 1986; Patchev et al., 2004). Injection of an ER-α agonist or an ER-β agonist in the perinatal period has been shown to decrease the neuronal cell density in the AVPV in female rats (Patchev et al., 2004), indicating that this reduction in neuronal cell density in the AVPV is an effect of estrogen mediated through binding to either ER-α or ER-β. The AVPV in female rats contains a greater numbers of tyrosine-hydroxylase (TH) mRNA-positive, dopaminergic neurons (Simerly et al., 1985) and Kiss1 mRNA-positive neurons (Kauffman et al., 2007) than the AVPV in male rats. Perinatal orchidectomy in males and perinatal treatment with testosterone in females resulted in an increase and decrease, respectively, in the number of TH mRNA-positive neurons in the AVPV (Simerly, 1989). In addition, perinatal treatment with TP in females decreased the number of Kiss1 mRNA-positive neurons (Kauffman et al., 2007).

## Investigation of sexual differentiation in the brain, by gonadal hormones and sex chromosomes using transgenic approaches in mice

There is accumulating evidence that a certain type of sexual differentiation in the brain is attributable to mechanisms that are independent of steroid hormones. The mouse model specific to this phenomenon called “four core genotypes” (FCG) was made possible by bioengineering techniques that created a mismatch between gonadal sex and chromosomal sex (XX vs. XY). This FCG model provides a breakthrough in the understanding of how sex chromosomes affect sexual differentiation in the brain. Based on observations in the FCG mouse model, the investigators reported that the latency and frequency of copulatory behavior are somewhat influenced by sex chromosomes (Park et al., 2008). However, sexual orientation seems to be mainly determined by differences in gonadal sex and subsequent secretion of sex steroids. This finding is generally consistent with the results of studies in which the sexual differentiation in the brain was examined after hormonal manipulation during the critical period. Indeed, the FCG model revealed that expression of progesterone receptor, which is inducible under the control of estrogen, is also dependent on gonadal sex (Wagner et al., 2004). On the other hand, aggression manifested in the form of attacks against intruders and parenting studied in pup-retrieval tests, have been reported to be regulated by chromosomal sex as well as by gonadal sex steroids (Gatewood et al., 2006). The aggression score, based on the proportion of mice that attacked intruders and the latency of attacking on first trial, was reported to increase in the presence of either testes or Y chromosome. By contrast, parental behavior, scored by latency to retrieve pups and number of pups retrieved, was low in the presence of either testes or Y chromosome. The sexual orientation of social behavior, including sniffing and play behavior in juvenile mice has been reported to be organized, at least in part, by the interaction between gonadal sex and chromosomal sex (Cox and Rissman, 2011). Therefore, neural circuits in the brain, responsible for social communications such as aggression, sniffing, and play behavior, may be differentiated not only by gonadal hormones, but also by the sex chromosome complement. In addition, the differentiation of neural circuits related to nociception, drug abuse, and autoimmune disease is related to the chromosomal sex, although the precise mechanisms by which chromosomal sex affects the neuronal circuits are not yet known. Taken together, the differentiation of core sexual behavior might be predominantly under the control of gonadal hormones, whereas the sex differentiation of various other aspects of physiology and behavior, including social communications might be determined, at least in part, by the interaction of gonadal hormones and sex chromosomes in mice.

## Effects of chromosomal sex on the structure and gene expression in the brain

The sexual dimorphism of midbrain dopaminergic neurons in rodents is reported to be controlled directly by chromosomal sex. Mice that have a Y chromosome, irrespective of their gonadal sex, have more dopaminergic neurons in their midbrain than mice that have only X chromosomes (Carruth et al., 2002). Expression of *Sry*, located on the Y chromosome in the male brain, has been reported to directly affect TH expression in the dopaminergic neurons of the substantia nigra (Dewing et al., 2006). In the lateral septum, on the other hand, both testosterone and the presence of a Y chromosome in male mice have been reported to increase the number of vasopressin neural fibers (De Vries et al., 2002). De Vries et al. revealed that XY males whose *Sry* gene was lost from the Y chromosome but had the heterotopic *Sry* transgene show a higher density of vasopressin fibers in the lateral septum than XX “males” with a heterotopic *Sry* transgene, whereas XY “females” whose *Sry* gene was lost from Y chromosome showed a higher density of vasopressin fibers in the area compared to the XX females (De Vries et al., 2002). These reports suggest that certain genes that are located on the Y chromosome, other than *Sry* affect the sexually dimorphic structure of the brain in mice. In association with such structural differences caused by sex chromosomes, expression of the genes located on X and Y chromosomes is also sexually dimorphic in the mouse brain (Xu et al., 2002; Dewing et al., 2003).

In addition, substantial differences in expression between sex-specific parental alleles on the X chromosome have been reported in the mouse brain (Gregg et al., 2010a). One study demonstrated that sex-specific imprinted genes whose expression differs between paternal and maternal alleles are mostly found in the hypothalamic area in the female brain (Gregg et al., 2010a), although early studies showed that maternal and paternal influence occurs in the cortex and in the hypothalamus, respectively (Allen et al., 1995; Keverne et al., 1996). On the other hand, paternal bias of autosomal genes in the brain was also reported (Gregg et al., 2010b). Understanding the epigenetic process that underlies the mechanism of parental bias would open up a new avenue of research on sex chromosomal effects in the brain.

## Gonadal hormone-dependent sexual differentiation in the avian brain

The Japanese quail is an animal model that has been used to examine how sex steroids determine the sexual differentiation in the brains of birds (Balthazart et al., 1983). The mating behavior of Japanese quail is sexually dimorphic: males strut and crow in front of the females and mount them (Adkins and Pniewski, 1978), whereas females never exhibit mounting behavior, even when injected with testosterone at adulthood (Adkins, 1975; Balthazart et al., 1983). It has been reported that in the adult male quail, estradiol produced by aromatization of testosterone in the brain induces male mounting behavior and that the testosterone metabolite 5-hydrotestosterone in the adult male induces strutting and crowing (Adkins and Pniewski, 1978; Balthazart et al., 1985). In contrast, exposure of male Japanese quail embryos to either testosterone or estrogen prior to day 12 of incubation resulted in significant reduction of mounting behavior at adult stage, indicating that actions of testosterone and estrogen at embryonic stage demasculinize male-type copulatory behavior at adulthood (Adkins-Regan, 1987). On the other hand, administration of an anti-estrogen agent to females prior to day 9 of incubation masculinizes their copulatory behavior (Adkins-Regan and Garcia, 1986). These results suggest that the order *Galliformes* and mammals are different in terms of the developmental effects of steroids. More specifically, the neuronal circuit related to copulatory behavior is masculinized in mammals by estrogen produced from testosterone in the brain, whereas it is feminized and de-masculinized in *Galliformes* by estrogen secreted from the ovary. As for plasma steroids in Japanese quail, it has been reported that from day 10 of incubation to hatching, estrogen concentrations are higher in females than in males, and conversely, testosterone concentrations are higher in males than in females (Ottinger et al., 2001). Therefore, the mechanism protecting the male quail brain from testosterone exposure has been postulated but is still under debate. Since a high activity of 5β-reductase has been reported in the embryonic male brain (Balthazart and Ottinger, 1984), this enzyme possibly metabolizes testosterone into 5β-dihydrotestosterone instead of E2 and protects the male brain from being de-masculinized by testosterone exposure (Balthazart and Ottinger, 1984). Taken together, these results suggest that endogenous estrogen secreted by the ovary is sufficient to differentiate the neuronal circuits related to copulatory behavior into the female-type in quail, but the precise mechanism by which the male brain escapes from feminization remains unclear.

## Sexual differentiation in the song control system in the avian brain

Song performance of the zebra finch is observed only in males and song-related nuclei including the nucleus hyperstriatum ventrale, pars caudale (HVc), and the nucleus robustus archistriatalis (RA) have been reported to be sexually dimorphic. The song performance is affected by developmental exposure of steroids. Double treatment, consisting of either estrogen or testosterone during hatching and testosterone at the adult stage, enables females to sing (Gurney and Konishi, 1980), indicating that the presence of estrogen in the brain during development can masculinize the song-related nuclei. Thus, there may be a difference between the actions of steroids on sexual behavior in *Galliformes* and on song performance of *Passeriformes* during development. Although the source of estradiol in the song control system during development of the zebra finch was long unknown, male brain slices containing the HVc and the RA regions have been found to produce more estrogen than corresponding female brain slices (Holloway and Clayton, 2001). This suggests that estrogen produced locally in the HVc and/or the RA contributes to masculinization of song-related nuclei. Indeed, various steroid-synthesizing enzymes are expressed in the zebra finch brain (Schlinger and Remage-Healey, 2012) and the same is true for the Japanese quail brain (Tsutsui, 2011). Studies performed by Remage-Healey et al. (2010, 2013) suggested that neurosteroids rapidly produced in the brain are important for social interaction via modulation of song production and perception of acoustic signals.

## Gonadal hormone-independent sexual differentiation of the avian brain

It has been suspected that genes located on the sex chromosome act in a cell-autonomous manner in brain cells to differentiate song-related circuits. Findings from a naturally-occurring gynandromorphic finch whose right and left sides show different sex genetics, demonstrated that the sexually dimorphic neural circuit for the song system is differentiated in part due to chromosomal sex. Most interestingly, the expression level of androgen receptor in the one half showed a masculine phenotype as compared to the other half, despite the identical influence of steroid hormones on both sides (Agate et al., 2003). Similar chromosomal sex influences on the sex difference of somatic tissues may also apply to *Galliformes*. The coloration, wattle, and leg spur of gynandromorphic chicken were observed to be different on the right and left, indicating that cell-autonomous sex determination in somatic cells occurs in *Galliformes* as well as in *Passeriformes* (Zhao et al., 2010).

## Investigation of avian sexual differentiation using a chimera in which the sex chromosomal set in the brain differs from that for other somatic tissues

To determine whether the sex of the brain affects brain function and behavior, chimeras were constructed, in which the brains of two embryos were switched. The pioneering work by Nicole Le Douarin showed that developmental fate of cells can be monitored by creating quail-chick chimeras (Le Douarin and Jotereau, 1975). By a surgical method using a microscalpel, the brain primordium of a chick embryo at 1.5 days after incubation of the egg could be replaced by that of a quail embryo (Balaban et al., 1988; Teillet et al., 1991). By applying a similar method, the male (female) brain could be replaced by the female (male) brain of conspecies in *Galliformes*.

The first study with chimeras that have a brain with different chromosomal sex was conducted in Japanese quail (Gahr, 2003). In this study, the forebrain primordia were switched between two embryos before gonadal differentiation. The results showed that the chimeras with female karyotype in the forebrain but a male karyotype in other tissues did not exhibit mounting and exhibited only rudimentary crowing behavior. Since the adult chimeras showed low plasma level of testosterone which is required for male-type copulatory behavior, their impaired reproductive behaviors were attributable to their lower testosterone level. The volume of the preoptic area (POA) which is known to be dependent on plasma testosterone level (Panzica et al., 1987) was also reported to be feminized in the chimeras (Gahr, 2003). Therefore, it is speculated that male-typical chromosomal set complement is required in the forebrain of the quail, so that gonadotropin regulation can maintain testicular function in males.

On the other hand, we recently analyzed chicken chimeras with a brain of different chromosomal sex (Maekawa et al., 2013). However, in contrast to the previously observed results in Japanese quail chimeras, we did not observe any abnormalities in male-typical copulatory behavior and spermatogenesis in the chicken chimeras that had a male karyotype in gonads and a female karyotype in their brain. Rather, sexual maturation was delayed and an irregular ovulatory cycle was exhibited in the chimeras that had a female karyotype in their gonads and male karyotype in their brains. This abnormality in sexual maturation and ovulatory cycle was not reported in the previous study conducted with Japanese quail. Since the baseline gonadotropin level in chicken chimeras that had a female karyotype in their gonads and male karyotype in their brains was comparable to that in female chickens, we hypothesized that irregular oviposition in the chimeras is caused by a timing mismatch of the gonadotropin surge due to the male-type chromosomal sex in the brain.

Meanwhile, we also found that overall sexually dimorphic behavior, judged based on the results of the open field test and tests of sexual behavior, was not influenced by brain chromosomal sex. This suggests that gonadal steroids may determine brain function related to overall sexual dimorphic behavior. In addition, the blood steroid level of the chicken chimeras was not affected by the sex of the brain, which was different from the sex of the remaining tissues; the sexual dimorphism of the BNSTp, a nucleus that is thought to be related to sexual identity in humans, was dependent on gonadal hormones.

Only two studies of brain chimeras, one in Japanese quail and another in chicken, have ever been conducted. Both studies showed that brain chromosomal sex directly affects reproductive physiology, albeit with substantial discrepancies when compared in the details. We speculated the following three possible explanations for these discrepancies of pathology, resulting from transplantation:

1. *Species difference*: A comparison between the genome of quail and chicken revealed that chromosome rearrangements may have occurred between these two *Galliformes* species over 35 million years ago (Kayang et al., 2006). A draft Japanese quail genome sequence was assembled by means of next-generation sequencing technology (Kawahara-Miki et al., 2013). The results suggested that the genomes of Japanese quail and chicken were closely related while being more distantly related to the genome of the zebra finch (Kawahara-Miki et al., 2013). However, genomic variation (Kawahara-Miki et al., 2013) and differences in reproductive physiology (Yoshimura, 2013) between Japanese quail and chicken have been found and such differences may affect pathology.
2. *Differences between the methods of transplantation*: In the study conducted on Japanese quail chimera, only the forebrain was transplanted, whereas the total brain primordium was transplanted in our study of chicken chimera. Indeed, our preliminary results in quail-chick transplantation revealed that the midbrain in which the dopaminergic neurons are known to show sexual dimorphism under sex chromosomal control in mammals was not exchanged by forebrain transplantation.
3. *Rejection*: In our study of chicken chimera, the female tissue transplanted into male bodies was rejected at the time of sexual maturation (Maekawa et al., 2013). Since the rejection was strictly sex combination-dependent, we speculated that the rejection was attributable to the expression of a female-specific antigen coded by gene(s) on the W chromosome. Daily injection of an immunosuppressant was necessary to evaluate behavior and physiology of chicken chimeras that had a female karyotype in the brain and male karyotype in the rest of their body. On the other hand, no rejection was reported in the study on Japanese quail. It is possible that a mild rejection occurred in Japanese quail chimeras that had a female karyotype in the forebrain and male karyotype in the rest of their body. In fact, we experienced that the partial transplantation of the brain primordium led to a mild rejection, which did not cause the death of the chicken chimera.

Additional studies of Japanese quail and chicken chimeras that are created by the same experimental protocol would be necessary to fully understand whether the fundamental rule of brain sexual differentiation exists in *Galliformes*.

## Possible similarities in sexual differentiation of behavior and physiology in mammals and birds

Finally, we describe possible similarities in sexual differentiation of behavior and physiology in mammals and birds (Table 1).

**Table 1:** **Possible similarities of sex differences in brain, physiology and behavior in mammals and birds**.

**Similarities**		**Typical literatures**
1	Sex differentiation of core sexual behavior is mainly induced by the exposure of gonadal hormones during development	Arnold and Chen, 2009; Maekawa et al., 2013 (also, 2–5)
2	The structure of BNST (M > F) is sexually differentiated mainly by the exposure of gonadal hormones during development	Tsukahara et al., 2011
3	Sex chromosomes in brain affects sexual dimorphism observed in communication such as song production in bird and social behavior in mouse	Agate et al., 2003; Cox and Rissman, 2011
4	Sex-biased local synthesis of neuroestrogen (M > F)	Hojo et al., 2009, 2014
5	Sexual differentiation of neuronal structures related to circadian timing could be affected by sex chromosomes in the brain	Kuljis et al., 2013

### Sex differences in sexual behavior and brain structures

The sexual orientation of murine copulatory behavior is mainly determined by exposure of mice to sex steroids during development. Similarly, the sexual orientation of chicken copulatory behavior, including mounting in males and adopting a receptive posture in females, has been found in our study to be determined by gonadal sex. Taken together, the above results suggest that overall sexual orientation of the copulatory behavior of both mammals and birds may be regulated by gonadal hormones. The brain nuclei related to sexual functions, namely the SDN-POA and BNST in mammals and the BNST in the chicken, are reported to be differentiated in a sexual dimorphic manner mainly by gonadal hormones. On the other hand, song performance in male zebra finches and sexual dimorphism observed in mice social behavior were reported to be affected by the sex chromosomes in the brain. Studies on a variety of species of mammals and birds may lead to a clear understanding of the precise interaction between such behaviors and the effect of sex chromosome in the brain or gonadal hormones.

### Neurosteroid production

**Figure 1:**
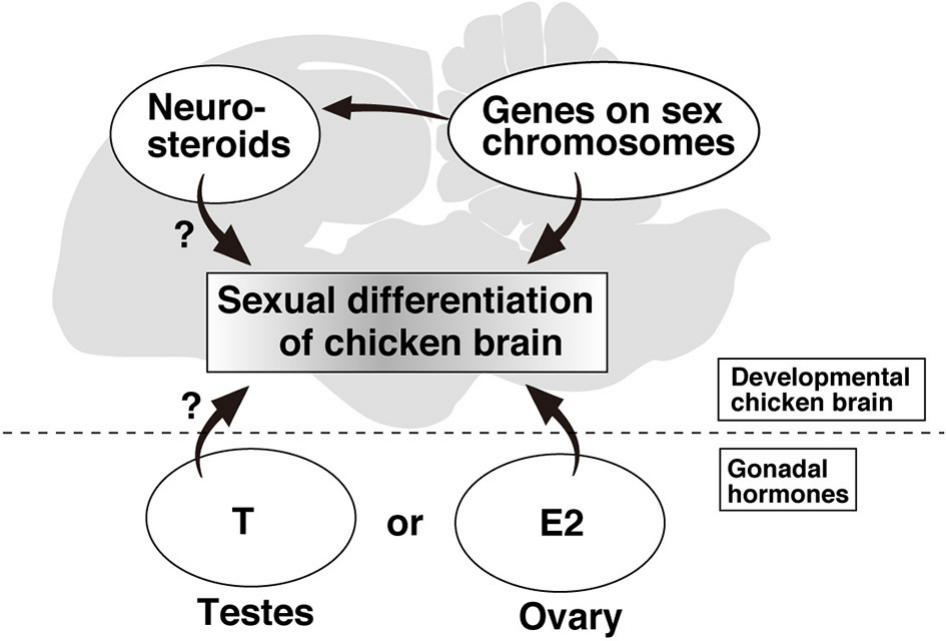
**Schematic diagram of the factors related to sexual differentiation of chicken brain**.

There is direct and indirect evidence that estrogen is locally produced in the songbird brain and that its concentration is higher in the specific brain nuclei in males. The estradiol level in whole brain, at embryonic day 21 and at 13 months of age has been found to be significantly higher in males than in females, even though the plasma estradiol level in females was higher (Maekawa et al., 2013). Moreover, chicken chimeras that had a female karyotype in their gonads and male karyotype in their brains had higher estradiol level in the brain relevant to male phenotype. Conversely, chimeras that had a female karyotype in their brains and male karyotype in their gonads had lower estradiol level relevant to female phenotype. These findings provide the first clear evidence that local estradiol production in the chicken brain is regulated by the chromosomal sex in the brain (Figure 1). Neuroestrogen has been reported to regulate the socio-sexual behavior in Japanese quail (Ubuka et al., 2014), although it remains uncertain whether sex difference of neuroestrogen is related to certain behavioral and physiological functions. In rat, the hippocampus has been shown to synthesize steroid hormones, which show sexual dimorphic concentrations (Hojo et al., 2004, 2014). The estradiol level of the female hippocampus at all stages of the estrous cycle was much lower than in the male (Hojo et al., 2009), and this finding is consistent with the results showing higher estradiol level in the brain of male zebra finch and chickens. The common mechanism underlying the sex-biased local synthesis of neuroestrogen in mammals and birds could be further elucidated by focused research on the expression of steroid-synthesizing enzymes.

### Circadian timing

Chicken chimeras that have a male karyotype in the brain and female karyotype in their gonads have an irregular ovulatory cycle (Maekawa et al., 2013). The circadian timing of ovulation was delayed in the chimera, indicating that the neural circuit responsible for the timing of ovulation may be differentiated under the influence of chromosomal sex of the brain. It has been reported that mice having the XX chromosomal complement have longer activity duration than mice having the XY chromosomal complement irrespective of their gonadal sex (Kuljis et al., 2013), suggesting that sex chromosomal effect in mouse brain affects the circadian biological clock. Thus, the sex differences of neuronal structures related to determination of or mediating circadian timing may provide an alternative target to elucidate sex-related brain function. Elucidation of the defect in circadian timing of ovulation found in chicken chimeras may provide new insights into the relationship between the biological clock and sex differences.

## Concluding remarks

In this review, we summarized the mechanisms underlying sexual differentiation of the behavior and physiology in mammals and birds. From a classical viewpoint, perinatal sex steroids secreted by the gonads differentiate the sex-specific behavioral and physiological functions as well as brain structures both in mammals and birds. However, recent studies suggest that brain sex chromosomes directly affect the sexual differentiation of certain types of behavior and physiology. Especially, our study using chicken chimeras revealed that brain sex chromosomes directly influence neurosteroid synthesis.

In the context of environmental sciences examining harmful effects of endocrine disruptors, several studies have been conducted to investigate how endocrine disruptors affect the production and secretion of steroid hormones, how they impair ligand-binding of steroid hormones to their receptors, and their effects on tissues. Based on the findings described in this review, the effects of endocrine disruptors on local steroid production in the brain should be considered as the focus for further investigation of the harmful effects of endocrine disruptors.

### Conflict of interest statement

The authors declare that the research was conducted in the absence of any commercial or financial relationships that could be construed as a potential conflict of interest.
